# Bis(dimethyl­malonato-κ^2^ *O*,*O*′)bis­[4-(4-pyridylamino-κ*N* ^4^)pyridinium]nickel(II) hexa­hydrate

**DOI:** 10.1107/S160053680803835X

**Published:** 2008-11-22

**Authors:** Gregory A. Farnum, Robert L. LaDuca

**Affiliations:** a Lyman Briggs College, Department of Chemistry, Michigan State University, East Lansing, MI 48825, USA

## Abstract

In the title compound, [Ni(C_5_H_6_O_4_)_2_(C_10_H_10_N_3_)_2_]·6H_2_O, divalent nickel ions situated on the crystallographic twofold axis are octa­hedrally coordinated by four O atoms from two dimethyl­malonate ligands in a 1,3-chelating mode and two N atoms from two protonated monodentate 4,4′-dipyridylamine mol­ecules. The mol­ecules link into chains *via* N—H⋯O hydrogen bonding mediated by protonated pyridyl groups. The chains form layer patterns *via* π–π stacking [centroid–centroid distance = 3.777 (2) Å] . Water mol­ecule hexa­mers are generated from the unligated water mol­ecules (three per asymmetric unit) by inversion centers at Wyckoff position *d*. These clusters are situated between the pseudolayers, providing hydrogen-bonding pathways that build up the three-dimensional structure.

## Related literature

For 4,4′-dipyridylamine (dpa) coordination polymers, see: Martin *et al.* (2007 ▶). For cobalt and nickel malonate dpa coordination polymers, see: Montney *et al.* (2008 ▶).
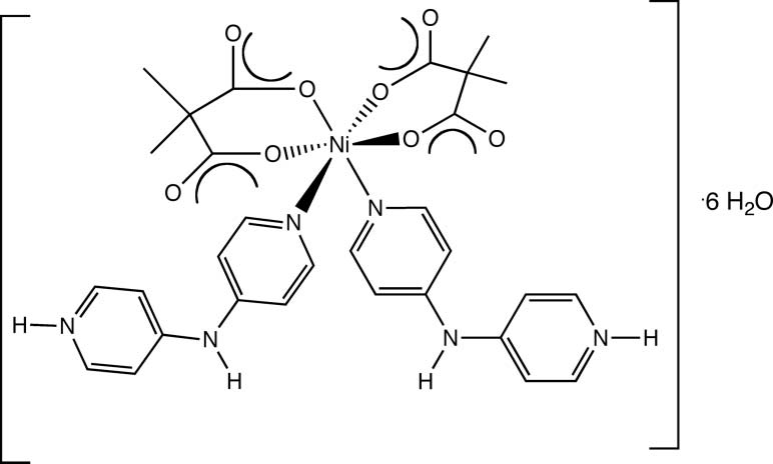


## Experimental

### 

#### Crystal data

[Ni(C_5_H_6_O_4_)_2_(C_10_H_10_N_3_)_2_]·6H_2_O
*M* *_r_* = 771.42
Monoclinic, 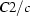

*a* = 18.428 (4) Å
*b* = 8.0473 (16) Å
*c* = 23.731 (5) Å
β = 97.96 (3)°
*V* = 3485.4 (12) Å^3^
*Z* = 4
Mo *K*α radiation
μ = 0.63 mm^−1^
*T* = 173 (2) K
0.30 × 0.30 × 0.10 mm

#### Data collection

Bruker SMART 1K diffractometer
Absorption correction: multi-scan (*TWINABS*; Sheldrick, 2007 ▶) *T*_min_ = 0.833, *T*_max_ = 0.939
49338 measured reflections
3998 independent reflections
3222 reflections with *I* > 2σ(*I*)
*R*_int_ = 0.079

#### Refinement

*R*[*F* ^2^ > 2σ(*F* ^2^)] = 0.054
*wR*(*F* ^2^) = 0.163
*S* = 1.09
3998 reflections
246 parameters
10 restraints
H atoms treated by a mixture of independent and constrained refinement
Δρ_max_ = 0.84 e Å^−3^
Δρ_min_ = −0.61 e Å^−3^

### 

Data collection: *SMART* (Bruker, 2006 ▶); cell refinement: *SAINT-Plus* (Bruker, 2006 ▶); data reduction: *SAINT-Plus* and *CELL-NOW* (Sheldrick, 2003 ▶); program(s) used to solve structure: *SHELXS97* (Sheldrick, 2008 ▶); program(s) used to refine structure: *SHELXL97* (Sheldrick, 2008 ▶); molecular graphics: *Crystal Maker* (Palmer, 2007 ▶); software used to prepare material for publication: *SHELXL97*.

## Supplementary Material

Crystal structure: contains datablocks I, global. DOI: 10.1107/S160053680803835X/pk2132sup1.cif

Structure factors: contains datablocks I. DOI: 10.1107/S160053680803835X/pk2132Isup2.hkl

Additional supplementary materials:  crystallographic information; 3D view; checkCIF report

## Figures and Tables

**Table 1:** Hydrogen-bond geometry (Å, °)

*D*—H⋯*A*	*D*—H	H⋯*A*	*D*⋯*A*	*D*—H⋯*A*
O1*W*—H1*WA*⋯O3*W*^i^	0.85	1.96	2.811 (4)	180
O1*W*—H1*WB*⋯O2*W*	0.840 (18)	2.05 (2)	2.870 (3)	166 (4)
O2*W*—H2*WA*⋯O3	0.840 (18)	1.904 (19)	2.741 (3)	174 (4)
O2*W*—H2*WB*⋯O4^ii^	0.844 (18)	1.95 (2)	2.751 (3)	158 (4)
O3*W*—H3*WA*⋯O1*W*	0.85	1.90	2.754 (4)	179
O3*W*—H3*WB*⋯O3^iii^	0.85	1.94	2.793 (3)	179
N2—H2N⋯O2*W*^iv^	0.866 (18)	2.16 (2)	2.985 (3)	158 (3)
N3—H3N⋯O2^v^	0.82 (4)	1.86 (4)	2.683 (3)	176 (4)

Symmetry codes: (i) 
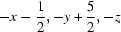
; (ii) 
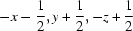
; (iii) 
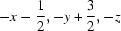
; (iv) 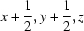; (v) 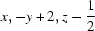.

**Fig. 1.**
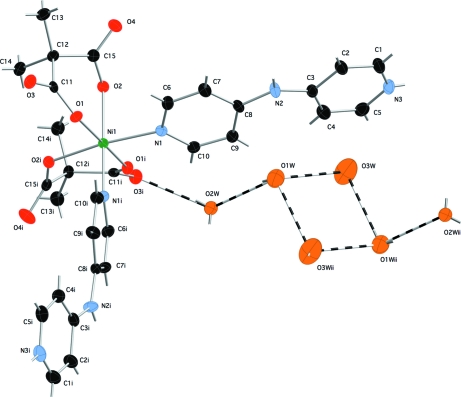
A full molecular unit of the title compound, along with hydrogen bonded water molecule hexamer, showing 50% probability ellipsoids and the atom numbering scheme. Hydrogen atom positions are shown as gray sticks. Hydrogen bonding interactions are shown as dashed lines. Color codes: green Ni, light blue N, red O, black C. Symmetry codes: (i) -x, y, -z + 1/2; (ii) -x - 1/2, -y + 5/2, -z

**Fig. 2.**
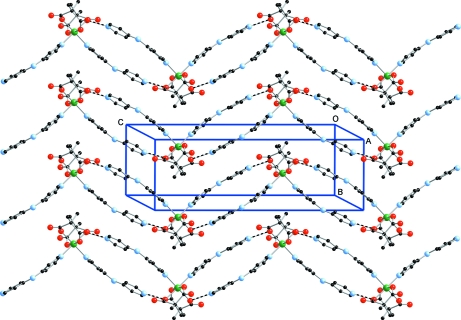
A single supramolecular layer in the title compound, formed from π–π stacking of hydrogen-bonded [Ni(dimethylmalonate)2(Hdpa)2]n supramolecular chains. Hydrogen bonding is indicated as dashed lines.

**Fig. 3.**
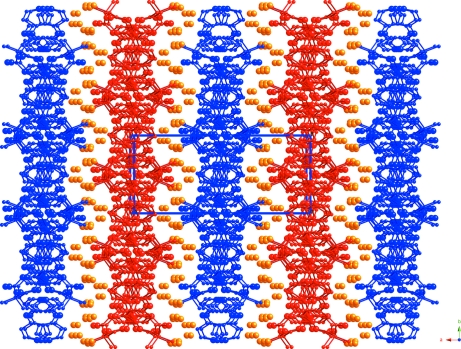
Packing diagram illustrating the AB layer stacking pattern, which forms the 3-D crystal structure of the title compound through hydrogen bonding between water molecules of crystallization and the amine groups of the Hdpa ligands. Individual pseudolayers are shown in blue and red. The oxygen atoms of the water molecules of crystallization are shown in orange.

## supplementary crystallographic information

### Comment

The dipodal tethering ligand 4,4'-dipyridylamine (dpa) has proven beneficial for the construction of coordination polymer solids with novel topologies (Martin *et al.*, 2007). Isostructural cobalt and nickel malonate dpa coordination polymers possess a three-dimensional 4^4^6^6^ sqp (square pyramidal) topology (Montney *et al.*, 2008). In an attempt to probe the effect of alkyl group substitution on coordination polymer structure by using dimethylmalonate, green crystals of the title compound were obtained.

The asymmetric unit of the title compound contains a nickel atom on a crystallographic two-fold axis, one dimethylmalonate dianion, one protonated Hdpa^+^ ligand and three water molecules of crystallization. Operation of the two-fold axis generates a neutral molecular complex, {[Ni(dimethylmalonate)_2_(Hdpa)_2_].6H_2_O}, in which the nickel atom is octahedrally coordinated (Fig. 1). The dimethylmalonate ligands bind in a 1,3-chelating fashion, each bridging two *cis* coordination sites. The Hdpa ligands are disposed in a *cis* fashion relative to each other.

Neighboring [Ni(dimethylmalonate)_2_(Hdpa)_2_ molecules are connected into supramolecular chain patterns, parallel to the *c* crystal direction, through hydrogen bonding between the protonated pyridyl termini of the Hdpa ligands and unligated dimethylmalonate oxygen atoms. These chains interact *via*π–π stacking between protonated pyridyl rings to form supramolecular layers oriented parallel to the *bc* crystal planes (Fig. 2). The supramolecular layers interact with each other by hydrogen bonding patterns between the dpa central amine groups or dimethylmalonate carboxylate groups and water molecules of crystallization to form the three-dimensional structure of the title compound (Fig. 3). The unligated water molecules themselves form a hydrogen bonded hexameric cluster centered on a cyclic tetrameric unit, as seen in Fig. 1. The centroids of the clusters rest on crystallographic inversion centers (Wyckoff position d).

### Experimental

All chemicals were obtained commercially. Nickel perchlorate hexahydrate (135 mg, 0.37 mmol) and dimethylmalonic acid (49 mg, 0.74 mmol) were dissolved in 3 ml water in a glass vial. A 1 ml aliquot of a 1:1 water–ethanol was carefully layered onto the aqueous solution, followed by 3 ml of an ethanolic solution of dpa (127 mg, 0.74 mmol). Green blocks of the title compound formed after 1 week.

### Refinement

All H atoms bound to C atoms were placed in calculated positions, with C—H = 0.95 Å and refined in riding mode with *U*_iso_ = 1.2*U*_eq_(C). The H atoms bound to O atoms were found *via* Fourier difference map, restrained at fixed positions or with O—H = 0.85 Å, and refined with *U*_iso_ = 1.2*U*_eq_(O). The H atoms bound to N atoms were found *via* Fourier difference map, restrained with N—H = 0.89 Å, and refined with *U*_iso_ = 1.2*U*_eq_(N).

### Crystal data

**Table:**

[Ni(C_5_H_6_O_4_)_2_(C_10_H_10_N_3_)_2_]·6H_2_O	*F*_000_ = 1624
*M**_r_* = 771.42	*D*_x_ = 1.470 Mg m^−^^3^
Monoclinic, *C*2/*c*	Mo *K*α radiation λ = 0.71073 Å
Hall symbol: -C 2yc	Cell parameters from 49338 reflections
*a* = 18.428 (4) Å	θ = 1.7–28.1º
*b* = 8.0473 (16) Å	µ = 0.63 mm^−^^1^
*c* = 23.731 (5) Å	*T* = 173 (2) K
β = 97.96 (3)º	Block, green
*V* = 3485.4 (12) Å^3^	0.30 × 0.30 × 0.10 mm
*Z* = 4	

### Data collection

**Table:**

Bruker SMART 1K diffractometer	3998 independent reflections
Radiation source: fine-focus sealed tube	3222 reflections with *I* > 2σ(*I*)
Monochromator: graphite	*R*_int_ = 0.079
*T* = 173(2) K	θ_max_ = 28.1º
ω scans	θ_min_ = 1.7º
Absorption correction: multi-scan(TWINABS; Sheldrick, 2007)	*h* = −20→24
*T*_min_ = 0.833, *T*_max_ = 0.939	*k* = −10→0
49338 measured reflections	*l* = −19→31

### Refinement

**Table:**

Refinement on *F*^2^	Secondary atom site location: difference Fourier map
Least-squares matrix: full	Hydrogen site location: inferred from neighbouring sites
*R*[*F*^2^ > 2σ(*F*^2^)] = 0.054	H atoms treated by a mixture of independent and constrained refinement
*wR*(*F*^2^) = 0.163	*w* = 1/[σ^2^(*F*_o_^2^) + (0.0996*P*)^2^ + 4.676*P*] where *P* = (*F*_o_^2^ + 2*F*_c_^2^)/3
*S* = 1.09	(Δ/σ)_max_ < 0.001
3998 reflections	Δρ_max_ = 0.84 e Å^−^^3^
246 parameters	Δρ_min_ = −0.60 e Å^−^^3^
10 restraints	Extinction correction: none
Primary atom site location: structure-invariant direct methods	

### Special details

**Table:**

Experimental. Reflection data were collected on a non-merohedrally twinned crystal. The twin law was determined with *CELL-NOW* (Sheldrick, 2003). The structure was solved and refined using reflections from only the major twin component, whose reflection file was generated using TWINABS (Sheldrick, 2007). Composite reflections belonging to both twin domains were omitted from the reflection list, causing the loss of 252 reflections from the major twin component data. The data set was still 99.9% complete to 2θ of 50°.
Geometry. All e.s.d.'s (except the e.s.d. in the dihedral angle between two l.s. planes) are estimated using the full covariance matrix. The cell e.s.d.'s are taken into account individually in the estimation of e.s.d.'s in distances, angles and torsion angles; correlations between e.s.d.'s in cell parameters are only used when they are defined by crystal symmetry. An approximate (isotropic) treatment of cell e.s.d.'s is used for estimating e.s.d.'s involving l.s. planes.
Refinement. Refinement of *F*^2^ against ALL reflections. The weighted *R*-factor *wR* and goodness of fit *S* are based on *F*^2^, conventional *R*-factors *R* are based on *F*, with *F* set to zero for negative *F*^2^. The threshold expression of *F*^2^ > σ(*F*^2^) is used only for calculating *R*-factors(gt) *etc*. and is not relevant to the choice of reflections for refinement. *R*-factors based on *F*^2^ are statistically about twice as large as those based on *F*, and *R*- factors based on ALL data will be even larger.

### Fractional atomic coordinates and isotropic or equivalent isotropic displacement parameters (Å^2^)

**Table:**

	*x*	*y*	*z*	*U*_iso_*/*U*_eq_	
Ni1	0.0000	0.68844 (6)	0.2500	0.01265 (17)	
O1	−0.10290 (10)	0.6794 (2)	0.20559 (8)	0.0169 (4)	
O1W	−0.18013 (13)	1.0666 (3)	0.04163 (11)	0.0386 (6)	
H1WA	−0.2030	1.1538	0.0492	0.046*	
H1WB	−0.198 (2)	0.986 (3)	0.0576 (16)	0.046*	
O2	−0.03894 (10)	0.5158 (2)	0.30453 (7)	0.0163 (4)	
O2W	−0.26118 (12)	0.8279 (3)	0.09768 (9)	0.0270 (5)	
H2WA	−0.2400 (19)	0.746 (3)	0.1144 (14)	0.032*	
H2WB	−0.2807 (19)	0.888 (4)	0.1206 (13)	0.032*	
O3	−0.20007 (10)	0.5600 (3)	0.15784 (8)	0.0207 (4)	
O3W	−0.2449 (2)	1.1445 (4)	−0.06678 (12)	0.0712 (11)	
H3WA	−0.2253	1.1212	−0.0331	0.085*	
H3WB	−0.2620	1.0821	−0.0944	0.085*	
O4	−0.14160 (11)	0.4853 (3)	0.34207 (8)	0.0277 (5)	
N1	0.02492 (12)	0.8724 (3)	0.19288 (9)	0.0151 (5)	
N2	0.08802 (13)	1.1902 (3)	0.06757 (10)	0.0199 (5)	
H2N	0.1351 (10)	1.203 (4)	0.0737 (15)	0.024*	
N3	0.00229 (14)	1.3886 (3)	−0.08748 (10)	0.0231 (5)	
H3N	−0.0118 (19)	1.422 (5)	−0.1199 (16)	0.028*	
C1	0.07385 (18)	1.3998 (4)	−0.06814 (12)	0.0262 (7)	
H1	0.1052	1.4516	−0.0902	0.031*	
C2	0.10115 (17)	1.3359 (4)	−0.01648 (12)	0.0236 (6)	
H2	0.1508	1.3467	−0.0031	0.028*	
C3	0.05471 (16)	1.2533 (4)	0.01689 (11)	0.0193 (6)	
C4	−0.01943 (16)	1.2421 (4)	−0.00520 (12)	0.0228 (6)	
H4	−0.0521	1.1883	0.0152	0.027*	
C5	−0.04357 (17)	1.3111 (4)	−0.05712 (13)	0.0251 (6)	
H5	−0.0930	1.3040	−0.0716	0.030*	
C6	0.09491 (14)	0.9063 (4)	0.18757 (11)	0.0186 (6)	
H6	0.1315	0.8551	0.2126	0.022*	
C7	0.11574 (15)	1.0124 (4)	0.14740 (11)	0.0193 (6)	
H7	0.1651	1.0319	0.1457	0.023*	
C8	0.06220 (15)	1.0909 (3)	0.10910 (11)	0.0169 (5)	
C9	−0.01058 (15)	1.0630 (4)	0.11588 (11)	0.0205 (6)	
H9	−0.0482	1.1167	0.0927	0.025*	
C10	−0.02595 (15)	0.9540 (4)	0.15780 (11)	0.0193 (6)	
H10	−0.0749	0.9364	0.1618	0.023*	
C11	−0.14996 (13)	0.5650 (3)	0.19920 (10)	0.0134 (5)	
C12	−0.14587 (14)	0.4205 (3)	0.24216 (11)	0.0159 (5)	
C13	−0.22274 (15)	0.3538 (4)	0.24762 (12)	0.0220 (6)	
H13A	−0.2462	0.3181	0.2110	0.033*	
H13B	−0.2513	0.4401	0.2618	0.033*	
H13C	−0.2187	0.2615	0.2735	0.033*	
C14	−0.10114 (16)	0.2814 (4)	0.21810 (12)	0.0211 (6)	
H14A	−0.1258	0.2466	0.1817	0.032*	
H14B	−0.0965	0.1887	0.2438	0.032*	
H14C	−0.0533	0.3226	0.2138	0.032*	
C15	−0.10720 (14)	0.4782 (3)	0.30049 (11)	0.0163 (5)	

### Atomic displacement parameters (Å^2^)

**Table:**

	*U*^11^	*U*^22^	*U*^33^	*U*^12^	*U*^13^	*U*^23^
Ni1	0.0109 (3)	0.0145 (3)	0.0122 (2)	0.000	0.00056 (16)	0.000
O1	0.0145 (9)	0.0167 (10)	0.0186 (9)	−0.0025 (7)	−0.0008 (7)	0.0019 (7)
O1W	0.0325 (13)	0.0370 (15)	0.0459 (15)	−0.0012 (11)	0.0042 (11)	0.0057 (11)
O2	0.0152 (9)	0.0195 (10)	0.0137 (9)	−0.0010 (7)	−0.0003 (7)	0.0010 (7)
O2W	0.0282 (12)	0.0304 (13)	0.0228 (11)	0.0086 (9)	0.0044 (9)	0.0049 (9)
O3	0.0183 (10)	0.0239 (11)	0.0180 (9)	−0.0021 (8)	−0.0049 (7)	−0.0008 (8)
O3W	0.108 (3)	0.062 (2)	0.0338 (15)	0.026 (2)	−0.0247 (16)	−0.0222 (14)
O4	0.0259 (11)	0.0408 (14)	0.0175 (10)	−0.0098 (10)	0.0063 (8)	−0.0048 (9)
N1	0.0149 (10)	0.0150 (11)	0.0157 (10)	−0.0010 (9)	0.0030 (8)	−0.0010 (9)
N2	0.0163 (11)	0.0258 (13)	0.0168 (11)	−0.0029 (10)	−0.0006 (9)	0.0077 (9)
N3	0.0323 (14)	0.0223 (13)	0.0134 (11)	0.0046 (11)	−0.0014 (10)	0.0017 (10)
C1	0.0328 (16)	0.0278 (17)	0.0188 (13)	0.0004 (13)	0.0062 (11)	0.0050 (12)
C2	0.0246 (14)	0.0294 (16)	0.0169 (13)	−0.0007 (12)	0.0031 (11)	0.0023 (11)
C3	0.0269 (15)	0.0175 (14)	0.0134 (12)	0.0017 (11)	0.0023 (10)	−0.0004 (10)
C4	0.0223 (14)	0.0276 (16)	0.0179 (13)	−0.0046 (12)	0.0004 (11)	−0.0013 (12)
C5	0.0241 (15)	0.0281 (16)	0.0218 (14)	0.0029 (12)	−0.0014 (11)	−0.0055 (12)
C6	0.0160 (13)	0.0210 (15)	0.0174 (12)	−0.0008 (11)	−0.0024 (10)	0.0023 (10)
C7	0.0140 (12)	0.0251 (15)	0.0182 (13)	−0.0057 (11)	0.0000 (10)	0.0007 (11)
C8	0.0206 (13)	0.0177 (14)	0.0126 (11)	−0.0039 (10)	0.0036 (10)	−0.0009 (10)
C9	0.0189 (13)	0.0230 (15)	0.0197 (13)	0.0042 (11)	0.0026 (10)	0.0040 (11)
C10	0.0157 (12)	0.0214 (15)	0.0209 (13)	0.0023 (11)	0.0027 (10)	0.0009 (11)
C11	0.0135 (12)	0.0154 (13)	0.0115 (11)	0.0013 (10)	0.0024 (9)	−0.0035 (9)
C12	0.0164 (12)	0.0153 (13)	0.0155 (12)	−0.0015 (10)	0.0002 (9)	0.0020 (10)
C13	0.0178 (13)	0.0239 (15)	0.0235 (14)	−0.0054 (11)	0.0007 (11)	0.0007 (11)
C14	0.0231 (14)	0.0172 (14)	0.0223 (14)	−0.0001 (11)	0.0005 (11)	−0.0022 (11)
C15	0.0181 (13)	0.0140 (13)	0.0161 (12)	0.0014 (10)	−0.0003 (10)	0.0029 (10)

### Geometric parameters (Å, °)

**Table:**

Ni1—O1	2.0392 (19)	C1—H1	0.9300
Ni1—O1^i^	2.0392 (19)	C2—C3	1.410 (4)
Ni1—O2^i^	2.0920 (19)	C2—H2	0.9300
Ni1—O2	2.0921 (19)	C3—C4	1.397 (4)
Ni1—N1^i^	2.100 (2)	C4—C5	1.368 (4)
Ni1—N1	2.100 (2)	C4—H4	0.9300
O1—C11	1.259 (3)	C5—H5	0.9300
O1W—H1WA	0.8506	C6—C7	1.373 (4)
O1W—H1WB	0.840 (18)	C6—H6	0.9300
O2—C15	1.284 (3)	C7—C8	1.396 (4)
O2W—H2WA	0.840 (18)	C7—H7	0.9300
O2W—H2WB	0.844 (18)	C8—C9	1.391 (4)
O3—C11	1.252 (3)	C9—C10	1.385 (4)
O3W—H3WA	0.8502	C9—H9	0.9300
O3W—H3WB	0.8499	C10—H10	0.9300
O4—C15	1.246 (3)	C11—C12	1.541 (4)
N1—C10	1.337 (3)	C12—C13	1.537 (4)
N1—C6	1.341 (3)	C12—C15	1.538 (4)
N2—C3	1.370 (3)	C12—C14	1.545 (4)
N2—C8	1.402 (3)	C13—H13A	0.9600
N2—H2N	0.866 (18)	C13—H13B	0.9600
N3—C1	1.338 (4)	C13—H13C	0.9600
N3—C5	1.338 (4)	C14—H14A	0.9600
N3—H3N	0.82 (4)	C14—H14B	0.9600
C1—C2	1.360 (4)	C14—H14C	0.9600
			
O1—Ni1—O1^i^	175.89 (10)	N3—C5—H5	119.2
O1—Ni1—O2^i^	91.75 (7)	C4—C5—H5	119.2
O1^i^—Ni1—O2^i^	85.51 (7)	N1—C6—C7	123.8 (2)
O1—Ni1—O2	85.51 (7)	N1—C6—H6	118.1
O1^i^—Ni1—O2	91.75 (7)	C7—C6—H6	118.1
O2^i^—Ni1—O2	96.77 (11)	C6—C7—C8	119.5 (2)
O1—Ni1—N1^i^	95.05 (8)	C6—C7—H7	120.2
O1^i^—Ni1—N1^i^	87.85 (8)	C8—C7—H7	120.2
O2^i^—Ni1—N1^i^	172.54 (8)	C9—C8—C7	117.2 (2)
O2—Ni1—N1^i^	86.81 (8)	C9—C8—N2	126.8 (2)
O1—Ni1—N1	87.85 (8)	C7—C8—N2	115.9 (2)
O1^i^—Ni1—N1	95.05 (8)	C10—C9—C8	118.8 (3)
O2^i^—Ni1—N1	86.81 (8)	C10—C9—H9	120.6
O2—Ni1—N1	172.54 (8)	C8—C9—H9	120.6
N1^i^—Ni1—N1	90.39 (12)	N1—C10—C9	124.3 (3)
C11—O1—Ni1	131.61 (17)	N1—C10—H10	117.9
H1WA—O1W—H1WB	108.1	C9—C10—H10	117.9
C15—O2—Ni1	121.78 (16)	O3—C11—O1	122.6 (2)
H2WA—O2W—H2WB	111 (3)	O3—C11—C12	117.2 (2)
H3WA—O3W—H3WB	131.1	O1—C11—C12	120.1 (2)
C10—N1—C6	116.2 (2)	C13—C12—C15	110.3 (2)
C10—N1—Ni1	123.44 (18)	C13—C12—C11	111.0 (2)
C6—N1—Ni1	120.21 (18)	C15—C12—C11	110.0 (2)
C3—N2—C8	132.4 (2)	C13—C12—C14	108.9 (2)
C3—N2—H2N	115 (2)	C15—C12—C14	110.3 (2)
C8—N2—H2N	112 (2)	C11—C12—C14	106.4 (2)
C1—N3—C5	120.8 (3)	C12—C13—H13A	109.5
C1—N3—H3N	118 (3)	C12—C13—H13B	109.5
C5—N3—H3N	121 (3)	H13A—C13—H13B	109.5
N3—C1—C2	120.5 (3)	C12—C13—H13C	109.5
N3—C1—H1	119.7	H13A—C13—H13C	109.5
C2—C1—H1	119.7	H13B—C13—H13C	109.5
C1—C2—C3	120.5 (3)	C12—C14—H14A	109.5
C1—C2—H2	119.8	C12—C14—H14B	109.5
C3—C2—H2	119.8	H14A—C14—H14B	109.5
N2—C3—C4	127.0 (3)	C12—C14—H14C	109.5
N2—C3—C2	115.8 (3)	H14A—C14—H14C	109.5
C4—C3—C2	117.2 (3)	H14B—C14—H14C	109.5
C5—C4—C3	119.4 (3)	O4—C15—O2	122.0 (2)
C5—C4—H4	120.3	O4—C15—C12	120.2 (2)
C3—C4—H4	120.3	O2—C15—C12	117.7 (2)
N3—C5—C4	121.5 (3)		
			
O1^i^—Ni1—O1—C11	18.9 (2)	C3—C4—C5—N3	0.3 (5)
O2^i^—Ni1—O1—C11	67.1 (2)	C10—N1—C6—C7	−2.8 (4)
O2—Ni1—O1—C11	−29.5 (2)	Ni1—N1—C6—C7	173.3 (2)
N1^i^—Ni1—O1—C11	−115.9 (2)	N1—C6—C7—C8	−0.3 (4)
N1—Ni1—O1—C11	153.9 (2)	C6—C7—C8—C9	3.3 (4)
O1—Ni1—O2—C15	−9.1 (2)	C6—C7—C8—N2	−176.7 (3)
O1^i^—Ni1—O2—C15	174.0 (2)	C3—N2—C8—C9	−17.2 (5)
O2^i^—Ni1—O2—C15	−100.3 (2)	C3—N2—C8—C7	162.8 (3)
N1^i^—Ni1—O2—C15	86.2 (2)	C7—C8—C9—C10	−3.2 (4)
N1—Ni1—O2—C15	18.1 (7)	N2—C8—C9—C10	176.8 (3)
O1—Ni1—N1—C10	15.1 (2)	C6—N1—C10—C9	2.9 (4)
O1^i^—Ni1—N1—C10	−167.8 (2)	Ni1—N1—C10—C9	−173.1 (2)
O2^i^—Ni1—N1—C10	107.0 (2)	C8—C9—C10—N1	0.1 (4)
O2—Ni1—N1—C10	−12.0 (7)	Ni1—O1—C11—O3	−157.24 (19)
N1^i^—Ni1—N1—C10	−80.0 (2)	Ni1—O1—C11—C12	20.7 (3)
O1—Ni1—N1—C6	−160.7 (2)	O3—C11—C12—C13	−32.9 (3)
O1^i^—Ni1—N1—C6	16.4 (2)	O1—C11—C12—C13	149.1 (2)
O2^i^—Ni1—N1—C6	−68.9 (2)	O3—C11—C12—C15	−155.2 (2)
O2—Ni1—N1—C6	172.2 (5)	O1—C11—C12—C15	26.7 (3)
N1^i^—Ni1—N1—C6	104.2 (2)	O3—C11—C12—C14	85.4 (3)
C5—N3—C1—C2	−1.6 (5)	O1—C11—C12—C14	−92.7 (3)
N3—C1—C2—C3	1.6 (5)	Ni1—O2—C15—O4	−127.2 (2)
C8—N2—C3—C4	5.0 (5)	Ni1—O2—C15—C12	53.7 (3)
C8—N2—C3—C2	−174.0 (3)	C13—C12—C15—O4	−8.5 (4)
C1—C2—C3—N2	178.5 (3)	C11—C12—C15—O4	114.2 (3)
C1—C2—C3—C4	−0.6 (4)	C14—C12—C15—O4	−128.8 (3)
N2—C3—C4—C5	−179.3 (3)	C13—C12—C15—O2	170.6 (2)
C2—C3—C4—C5	−0.3 (4)	C11—C12—C15—O2	−66.6 (3)
C1—N3—C5—C4	0.6 (5)	C14—C12—C15—O2	50.3 (3)

Symmetry codes: (i) −*x*, *y*, −*z*+1/2.

### Hydrogen-bond geometry (Å, °)

**Table:**

*D*—H···*A*	*D*—H	H···*A*	*D*···*A*	*D*—H···*A*
O1W—H1WA···O3W^ii^	0.85	1.96	2.811 (4)	180
O1W—H1WB···O2W	0.840 (18)	2.05 (2)	2.870 (3)	166 (4)
O2W—H2WA···O3	0.840 (18)	1.904 (19)	2.741 (3)	174 (4)
O2W—H2WB···O4^iii^	0.844 (18)	1.95 (2)	2.751 (3)	158 (4)
O3W—H3WA···O1W	0.85	1.90	2.754 (4)	179
O3W—H3WB···O3^iv^	0.85	1.94	2.793 (3)	179
N2—H2N···O2W^v^	0.866 (18)	2.16 (2)	2.985 (3)	158 (3)
N3—H3N···O2^vi^	0.82 (4)	1.86 (4)	2.683 (3)	176 (4)

Symmetry codes: (ii) −*x*−1/2, −*y*+5/2, −*z*; (iii) −*x*−1/2, *y*+1/2, −*z*+1/2; (iv) −*x*−1/2, −*y*+3/2, −*z*; (v) *x*+1/2, *y*+1/2, *z*; (vi) *x*, −*y*+2, *z*−1/2.
